# Protocol for a systematic review of screening tools for fear of recurrent illness in common life-threatening diseases

**DOI:** 10.1186/2046-4053-4-10

**Published:** 2015-03-19

**Authors:** Jenny Jones, Paul Kane, Rob Polson, Stephen J Leslie, Nicholas J Hulbert-Williams, Sébastien Simard, Gozde Ozakinci, Gill Hubbard

**Affiliations:** School of Nursing & Midwifery, Plymouth University, Plymouth, PL4 8AA UK; School of Health Sciences, University of Stirling, Old Perth Road, Inverness, IV2 3JH UK; Highland Health Sciences Library, Centre for Health Science, Old Perth Road, Inverness, IV2 3JH UK; Highland Heartbeat Centre, Cardiology Unit, Raigmore Hospital, Inverness, IV2 3UJ USA; Chester Research Unit for the Psychology of Health, University of Chester, Parkgate Road, Chester, CH1 4BJ UK; Institut Universitaire de Cardiologie et de Pneumologie, Hospital Laval, Québec, Canada; University of St Andrews, School of Medicine, St Andrews, KY16 9TF UK

**Keywords:** Fear of recurrence, Myocardial infarction, Cancer, Stroke, Acute coronary syndrome, Psychometric testing, Screening, Questionnaires

## Abstract

**Background:**

A myocardial infarction (MI) (‘heart attack’) can be intensely stressful, and the impact of this event can leave patients with clinically significant post-MI stress symptoms. Untreated stress can make heart disease worse. Few tools are available that screen for specific thoughts or beliefs that can trigger post-MI stress responses. In other life-threatening illnesses, fear of recurrence (FoR) of illness has been identified as a key stressor, and screening tools have been developed to identify this. The aim of this review is to identify FoR screening tools used in other common life-threatening diseases that report on the development of the tool, to assess if there are any that can be adapted for use in MI survivors so that those with high levels of FoR can be identified and helped.

**Methods/Design:**

The review will evaluate full FoR screening tools and methods of measurement used in common life-threatening disease clinical populations. The Campbell and Cochrane Libraries, Cumulative Index of Nursing and Allied Health Literature (CINAHL), PsycINFO, MEDLINE, Embase, Applied Social Sciences Index and Abstracts (ASSIA), Published International Literature on Traumatic Stress (PILOTS), Social Services Abstracts, Sociological Abstracts, Web of Knowledge, Health and Psychosocial Instruments and SCOPUS databases will be searched for relevant studies published from database inception. Reference lists and published reviews/meta-analyses will also be searched. All titles and abstracts will be screened and relevant full-text versions retrieved by two reviewers, who will then extract all the data. Each will independently review all data extracted by the other. Selected studies will also be assessed by two independent researchers using the COnsensus-based standards for the Selection of health status measurement INstruments (COSMIN) checklist and other quality criteria. This will be done to evaluate the degree to which their measurement properties meet the standards for good methodological quality. Disagreement will be resolved through consensus.

**Discussion:**

Untreated post-MI stress has a considerable psychological and physical impact on MI survivors. Therefore, there is a critical need to develop a screening tool to identify fear of recurrent MI so that those affected can be identified and directed to appropriate support interventions. This proposed research will enable a tool to be developed and adapted for use in the MI survivor patient population.

**Systematic review registration:**

PROSPERO: CRD42014010500

## Background

In the UK, there are over 100,000 incidences of myocardial infarctions (MI) every year, with a post-MI 30-day discharge survival rate of over 90% [1]. Scotland has one of the highest rates of MI in the world, despite a reported 25% reduction in incidence between 2000 and 2009 [2]. Over the last 15 years, specific interventions (coronary angioplasty, drugs, devices and cardiac rehabilitation) have considerably reduced MI-related mortality rates [3, 4].

Yet despite the improvements in treatment strategies, the experience of having an MI can be intensely stressful and frightening for many patients and the psychological impact of this event can be long lasting [5–8]. Clinically significant post-MI stress symptoms are thought to be present in up to 12.5% (one in eight) patients, and evidence indicates that the post-trauma stress may increase patients risk for subsequent cardiac events and mortality [9]. Even transient stress and anxiety appear to be associated with poorer longer-term outcomes [10] and short-term cardiovascular post-stress recovery rates [11]. Moreover, even cardiac patients with less clinically obvious, mild to moderate low mood, stress and anxiety are at risk of adverse outcomes, with evidence indicating that it is not simply the case (as has been argued in the past), that these elements merely co-exist alongside the pathophysiological effects, but rather that these mild to moderate elements and adverse cardiovascular reactions may indeed share a common (but as yet unidentified) underlying pathophysiological mechanism [12, 13]. Therefore, screening for modifiable psychological triggers of stress in post-MI patients to identify patients at risk is crucial to improving longer-term physical and psychological recovery. However, to date, few tools are available which allow cardiology service providers to screen and identify MI survivors for specific thoughts or beliefs that can trigger modifiable stress responses. In contrast, in other life-threatening illnesses such as cancer, ‘fear of illness recurrence’ has been identified as a key trigger of stress in patients and screening tools to identify this specific stressor have been developed [14–17].

Given the risk and impact of untreated post-MI stress, the development of a similar screening tool which can identify fear of recurrent MI in post-MI patients is therefore critical. In order for such a tool to be developed, it is necessary to identify and evaluate the quality of screening tools used to detect fear of recurrent illness in other common life-threatening diseases to find out if any existing fear of recurrence (FoR) tools can be adapted for use in post-MI patients. This evaluation will inform the development of a theory and evidence-based screening tool which can assess fear of recurrence in MI survivors to enable patients at risk to be directed to appropriate support and intervention. This proposed systematic review of the fear of recurrent illness literature aims to do just this.

### Study aims

The primary aim is to identify any fear of recurrent illness screening tools used in cancer, stroke, asthma and acute coronary syndrome patient populations and assess the quality of each tool to assess if there are any tools that can be adapted for FoR MI screening in MI survivors.

The secondary aim is to look at psychometric characteristics of FoR screening tools so that adaptations are only made to good quality and well-developed tools.

## Methods/Design

### Design

The review will evaluate FoR screening tools and methods of measurement in different clinical populations (cancer, stroke, asthma and acute coronary syndrome). Acute coronary syndrome encompasses a range of unstable coronary artery disease ranging from unstable angina to myocardial infarction [18]. These clinical populations are chosen because they are the most common life-threatening diseases in the UK, and survival from first occurrence and primary treatment has improved [19–22], but there is still risk of recurrence. Thus, FoR screening tools are important for these clinical populations.

### Strategy

An evidence synthesis of literature focusing on FoR in the most common life-threatening diseases in the UK will be conducted. Although existing literature identifies FoR as a common problem in cancer survivors, to date, there appears to be a lack of consensual definition of the phenomenon in the existing research [17]. Furthermore, the theoretical concept of FoR is currently unexplored in the MI survivor population. Therefore, for the purpose of this review, which is to examine the psychometric characteristics of FoR screening tools and assess potential transferability of FoR items, domains and constructs to a different survivor population, only FoR screening tools with >3 items will be included. Tools with less than three items tend to psychometrically unstable as with fewer items, cross-validation of factor structures becomes difficult [23]. Also, more comprehensive tools (>3 items) should offer greater sensitivity and granularity for the purposes of assessing potential transferability of meaningful and clinically relevant domains and items to a different survivor population. Sub-scales will be excluded as in existing FoR research, understanding of what existing sub-scales are measuring may be limited at this time, particularly as the most widely used sub-scale screening tools used for measuring patients’ point of view are typically derived from earlier longer versions of the same tool [24] or have reduced complex phenomenon to single items and domains without clear rationale for the non-standardised characteristics [25]. And as shorter versions typically reduce the original multiple items within a domain to single items, sub-scales cover a narrower range of precision and concept sensitivity because of this aggregation [26].

Systematic searches will be conducted across relevant health databases (Campbell and Cochrane Library, Cumulative Index of Nursing and Allied Health Literature (CINAHL), PsycINFO, MEDLINE, Embase, ASSIA, PILOTS, Social Services Abstracts, Sociological Abstracts, Web of Knowledge, Health and Psychosocial Instruments and SCOPUS) published in peer-reviewed journals from database inception (Table 1). Reference lists and published reviews/meta-analyses will also be searched. Studies involving the development of fear of illness recurrence screening or measurement tools will be evaluated. All titles and abstracts will be screened and relevant full-text versions retrieved by two reviewers.

**Table 1:** **Example search using subject headings**

	Search strategy
1	exp neoplasms/(2693021)
2	exp recurrence/(165159)
3	exp Neoplasm Recurrence, Local/(86083)
4	exp asthma/(109453)
5	exp stroke/(91990)
6	exp acute coronary syndrome/(8195)
7	2 or 3 (250583)
8	1 or 4 or 5 or 6 (2897701)
9	7 and 8 (116674)
10	exp emotions/(176489)
11	9 and 10 (261)
12	(cancer: or neoplasm: or oncol: or asthm: or stroke or strokes or acute coronary syndrom: or cardio: or cardia: or coronary).af. (4339611)
13	(recur: or rec-cur: or reocur: or re-ocur: or relaps: or reappear: or re appear).af. (585938)
14	(fear: or worr: or anx: or distress: or emotion: or apprehens: or dread or panic:).af. (411973)
15	12 and 13 and 14 (3417)
16	((questionnair: or scal: or screen: or psychometric:) and (valid: or reliab:)).af. (151160)
17	15 and 16 (113)
18	17 not 11 (86)

Database: Ovid MEDLINE(R) 1946 to Present with Daily Update.

Example search using keywords: Strategy used in ASSIA/PILOTS/Social Services Abstracts and Sociological Abstracts.

(cancer* OR neoplasm* OR oncol* OR asthm* OR stroke OR strokes OR acute coronary syndrom* OR cardio* OR cardia* OR coronary) AND (recur* OR rec-cur* OR reocur* OR re-ocur* OR relaps* OR reappear* OR re appear) AND (fear* OR worr* OR anx* OR distress* OR emotion* OR apprehens* OR dread OR panic*) AND (questionnair* OR scal* OR screen* OR Psychometric*) AND (valid* OR reliab*).

Strategy used in the non-structured databases.

(Myocardial infaction*) and (recur* or rec-cur* or reocur* or re-ocur* or relaps* or reappear* or re appear*) and (fear* or worr* or anx* or distress* or emotion* or apprehens* or dread or panic*).

### Inclusion criteria

All titles and abstracts will be screened and relevant full-text versions retrieved and assessed by two reviewers (Table 2). The following inclusion criteria will be applied: 1) studies that report on the development of screening tools used to measure fear of recurrent illness in life-threatening diseases (must include >3 items); 2) the study population should be adults diagnosed with cancer, stroke, asthma or acute coronary syndrome over 18 years of age; and 3) published in a peer-reviewed journal. Disagreements will be resolved through consensus, and the level of agreement will be reported and limitations acknowledged. All exclusion decisions will be reported.

**Table 2:** **Inclusion selection form**

Inclusion selection form	
1. Paper number	
2. First author	
3. Reviewer	
Q1. Does the study report on the development of a screening tool used to screen for fear of recurrent illness?	Yes ⎕ Go to Q2. No. Reject study.
Q2. Does the screening tool include > 3 items measuring fear of recurrent illness?	Yes ⎕ Go to Q3. No. Reject study.
Q3. Is the population adult patients in cancer, stroke, asthma or acute coronary syndrome?	Yes ⎕ Go to Q4. No. Reject study.
Q4. Is the study published in a peer-reviewed journal between 1946 - present?	Yes ⎕ Retain study. No. Reject study.

### Data extraction

A data extraction form will standardise the data extrapolated from each paper (Table 3). As health measurement instrument development lacks consensus at present and often includes different ways to measure a given construct, screening tools can vary widely in content, method and quality [27]. This means the fragmented health measurement literature is often non-comparable. Therefore, for the purposes of this review, we will adopt a ‘holistic’ approach to contrasting and comparing identified relevant tools, and narratively report on relevant quality or methodological properties relevant to the aim of this review. This approach will take into account quality measurements so that useful comparisons of quality can be made. In addition, design data will be extracted and summarised so that adaptations are only made to good quality and well-developed tools. Two researchers will extract data independently, then each will review all data extracted by the other and agree, through consensus, the accuracy and completeness of the data. Where consensus is difficult to reach, a third researcher will be consulted to reach agreement.

**Table 3:** **Data extraction items (in addition to COSMIN data extracted and reported)**

Descriptive information	Screening tool characteristics
Screening tool	Number of items
Clinical population	Number and types of domains
Sample size	Theoretical/conceptual framework
Disease stage	Response format/scale design
Country and setting	Tool development process
Type of instrument (e.g., PRO)	Validity (types of texts/results)
	Reliability
	Response rates
	Utility in administration and interpretation (including ceiling and floor scores)
	Range and number of raters required for scoring and analysis
	Mode of administration (e.g., self-completed)

### Study quality

**Figure 1:**
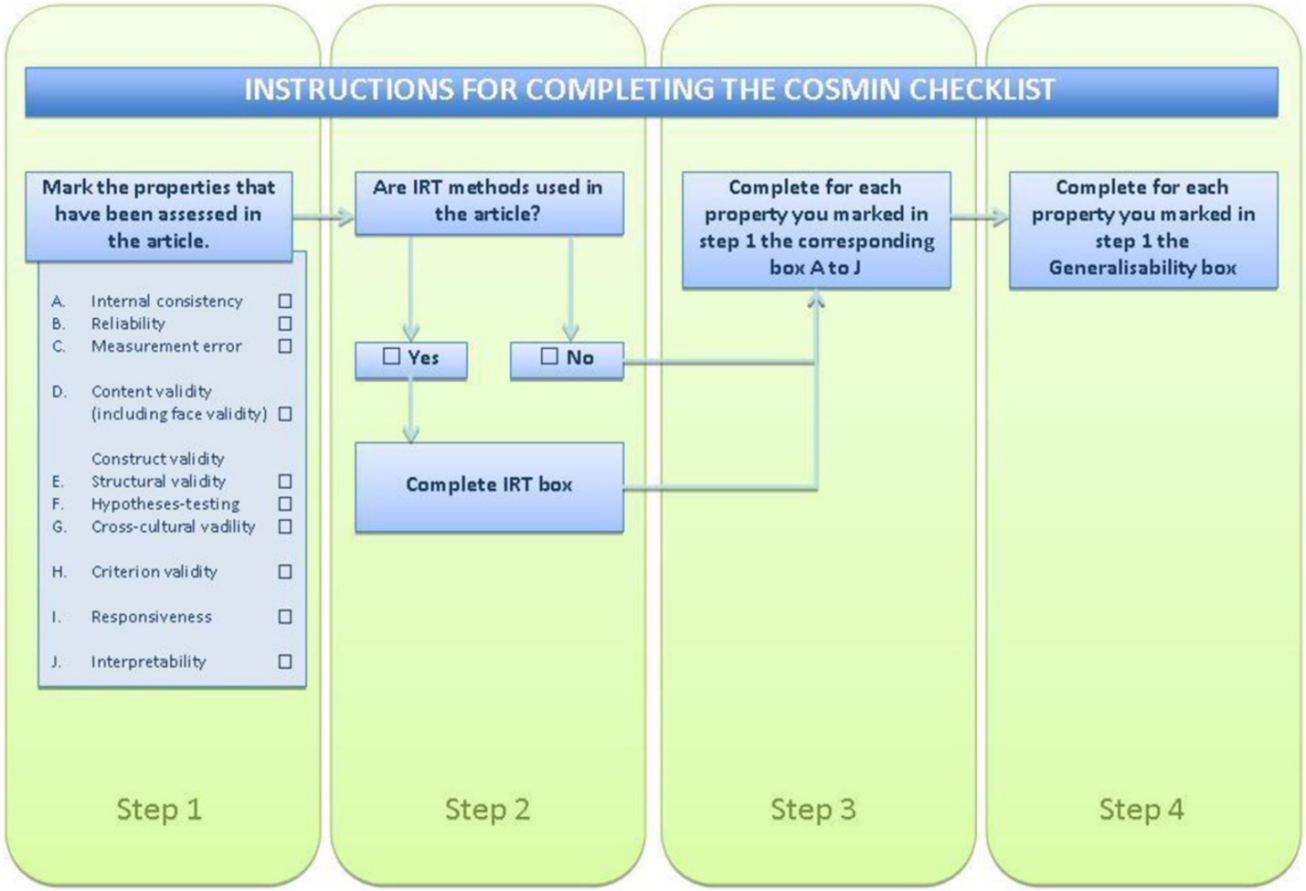
**The four-step procedure to complete the COSMIN checklist.**

Selected studies will also be assessed by two independent researchers using the COnsensus-based Standards for the selection of health status measurement INstruments (COSMIN) checklist to evaluate the degree to which their measurement properties meet the standards for good methodological quality. Disagreement will be resolved through consensus. The COSMIN checklist contains standards for design requirements and preferred statistical methods of studies on the measurement properties of health measurement instruments. The checklist can be used to determine if a study on measurement properties meets the standards for good methodological quality and has been developed by international experts in the field of health status measurement [28]. It is designed to evaluate the methodological quality of studies that report on psychometric properties for inclusion in a systematic review, even if the studies have conducted different validity and reliability tests. Each item of the COSMIN checklist data extraction form offers four possible response options (‘excellent’, ‘good’, ‘fair’, ‘poor’) in relation to methodological quality and the score obtained by taking the lowest rating of any item (Figure 1) [29]. This will allow us to report on the overall methodological quality of each study included in the review if sufficient data is reported in each publication.

### Data analysis

As study methods and development processes are anticipated to be heterogeneous, pooling of measurement tool properties is not possible. Therefore, synthesis of data will be primarily reported in words and text where appropriate, to summarise and explain the findings and content of multiple studies in narrative format. This synthesis will be based on the general framework and tools outlined in the ESRC Guidance on the Conduct of Narrative Synthesis in Systematic Reviews [30]. Relevant extracted study data including COSMIN ratings will be tabulated in a hierarchical rating of quality and individual instruments will be categorised in terms of relevant and comparable design features or characteristics [31].

## Discussion

Given the risk and consequences of untreated post-MI stress, the impact of this planned systematic review of fear of recurrent illness screening tools in common life-threatening diseases is expected to be high, as there is currently no fear of recurrent MI screening tool available. Therefore, there is a critical need to develop a screening tool to identify fear of recurrent MI in the MI survivor population so that post-MI patients with this fear can be identified and directed to appropriate support interventions. This proposed research will provide the best possible evidence to provide a foundation for the development of a fear of recurrent MI screening tool, by systematically and ‘holistically’ evaluating which fear of recurrence tools work, why they work and for whom. Thus, this research will enable an evidence and theory-based screening tool to be developed and adapted for use in the MI survivor patient population. This systematic review is timely and will make a valuable contribution to improving post-MI patient care.

## Abbreviations

MI: Myocardial infarction
ACS: Acute coronary syndrome
FoR: Fear of recurrence
FoRMI: Fear of recurrent myocardial infarction.
